# Surviving Allchar: arsenic and thallium tolerance and distribution in *Viola* metallophytes

**DOI:** 10.1093/aob/mcaf166

**Published:** 2025-07-19

**Authors:** Ksenija Jakovljević, Mirko Salinitro, Katerina Bačeva Andonovska, Tomica Mišljenović, Dennis Brueckner, Antony van der Ent

**Affiliations:** Department of Ecology, Institute for Biological Research Siniša Stanković, National Institute of the Republic of Serbia, University of Belgrade, Belgrade 11108, Serbia; Laboratory of Genetics, Wageningen University and Research, Wageningen 6708 PB, The Netherlands; Research Center for Environment and Materials, Macedonian Academy of Sciences and Arts, Skopje 1000, North Macedonia; Faculty of Biology, Institute of Botany and Botanical Garden, University of Belgrade, Belgrade 11000, Serbia; Deutsches Elektronen-Synchrotron DESY, Hamburg 22607, Germany; Laboratory of Genetics, Wageningen University and Research, Wageningen 6708 PB, The Netherlands; Université de Lorraine, INRAE, LSE, Nancy F-54000, France

**Keywords:** thallium, arsenic, hyperaccumulator, synchrotron-based micro-X-ray fluorescence analysis, *Viola*

## Abstract

**Background and Aims:**

*Viola tricolor* subsp. *macedonica* and *Viola arsenica* are metallophytes from the abandoned Allchar mine site in the Republic of North Macedonia, an area extremely enriched in thallium and arsenic, with up to 5750 and 12 800 mg kg^−1^ in the soil, respectively. This study aimed to infer tolerance mechanisms evolved in the two *Viola* species by analysis of the tissue-level distribution of arsenic and thallium.

**Methods:**

Seedlings of *V. tricolor* subsp. *macedonica* and *V*. *arsenica* were grown under different thallium and arsenic treatments in hydroponics. Synchrotron-based micro-X-ray fluorescence (µXRF) analysis was used to elucidate elemental distribution in hydrated plant organs and tissues.

**Key Results:**

Plants dosed with increasing concentrations of arsenic and thallium had higher accumulation of these elements, especially in the roots. In *V. arsenica*, thallium mainly accumulated in the shoots, with the mature leaves being the main site of deposition. In the leaves of *V*. *tricolor* subsp. *macedonica*, the highest thallium concentrations occured around the stomata.

**Conclusions:**

Foliar accumulation of thallium is the main tolerance strategy in *V. arsenica*, whereas the limited translocation into the shoot and potentially excreting excess thallium through the stomata in *V. tricolor* subsp. *macedonica* appears to be an important mechanism for survival in the extremely toxic habitat at the Allchar site.

## INTRODUCTION

Toxic metalliferous soils have been recognized as a driving force for plants to evolve and survive (Brady *et al.,* 2005). Although many plant species fail to adapt, some are able to thrive in highly edaphically stressful, but less competitive environments, through the evolution of various internal detoxification mechanisms (Murawska-Wlodarczyk *et al.*, 2022). These plant species have evolved hypertolerance to excessive concentrations of metals or metalloids in the soil on which they grow by either excluding metal(loids) (by highly restricted uptake or retention at the root level) or, more rarely, by accumulating metal(loids) in the above-ground shoots (Baker, 1981; Verbruggen *et al.*, 2009). At the extreme end of the range of the accumulator type species are the so-called hyperaccumulators with foliar concentrations of metal(loids) two to three orders of magnitude higher than in plants from non-metalliferous soils and at least one order of magnitude higher than in the leaves of plants from the metal-rich soils (van der Ent *et al.*, 2013). The study of hyperaccumulation is especially attractive in extremely metal-enriched areas, such as active and abandoned mines and their tailings, because of the suite of conditions plants must tolerate to survive there (Conesa *et al.*, 2006; Stefanowicz *et al.*, 2014; Wójcik *et al.*, 2014; Kasemodel *et al.*, 2019).

A particularly interesting case study is the abandoned Allchar mine site, located in the southern part of the Republic of North Macedonia on the slopes of Mount Kožuf, whose remarkable mineralogy with large deposits of thallium (Tl) and arsenic (As ores, makes it unique in the world (Boev *et al.*, 2012). These two elements are well known for their toxicity, with the lethal threshold for most plants being 20 mg kg^−1^ for Tl and 80 mg kg^−1^ for As in their leaves (Krämer, 2010). In humans, gastrointestinal disorders can be caused by acute poisoning and polyneuritis by chronic exposure to Tl, whereas encephalopathy and peripheral neuropathy can occur on exposure to As (Zhou *et al.*, 2021). Thallium and As originate from both geological and anthropogenic sources. Besides sulphidic ore deposits (with minerals including lorandite, ellisite, stibnite, pyrite, sphalerite, *etc*.) and potassiumrich minerals, such as feldspar (Belzile and Chen, 2017) that are natural sources of Tl, the main dispersed anthropogenic sources of Tl are various types of metallurgical industries, waste incineration and cement production (Wei *et al.*, 2020; Migaszewski and Gałuszka, 2021; Şevik *et al.*, 2024). Although there are also large concentrated Tl ore deposits in France, Italy, Switzerland, Poland and China (Migaszewski and Gałuszka, 2021), the Allchar deposit is unique with extremely rich Tl mineral occurenceswith lorandite and 12 other Tl minerals (Boev et al., 2001-2002) and soil concentrations of ≤5750 mg kg^−1^ Tl (Jakovljević *et al.*, 2025). Apart from Tl, As is highly enrichedat the Allchar site. To date, at least 568 minerals are known to contain As, and weathering of rocks is the most important natural source of this element (Punshon *et al.*, 2017). Depending on the bedrock characteristic and environmental conditions, the As concentration in soil can be high, up to 12 800 mg kg^−1^ at the Allchar site (Bačeva Andonovska *et al.*, 2021) and up to 20 700 mg kg^−1^ in gold mine tailings in New Scotia (Wong *et al.*, 1999). In contrast, anthropogenic sources of As are much more diverse and include mining, coal combustion, pesticides, fertilizers (both natural and artificial) and wood preservatives (Punshon *et al.*, 2017; Çobanoğlu and Kulaç, 2025).

The uptake and distribution of elements in plants depends on many factors and is largely species specific. Understanding these phenomena is particularly important for highly toxic elements, such as Tl and As, because they are readily taken up owing to their chemical similarity to essential elements. Monovalent Tl^+^ has been posited to use the uptake channels and distribution pathways of potassium ions (K^+^) and interferes with its metabolic processes (Corzo Remigio *et al.*, 2022*a*), whereas for As arsenate (AsO_4_^3-^) and phosphate (PO_4_^3-^) are chemical analogues (Pita-Barbosa *et al.*, 2019). The hyperaccumulation of Tl and As is an extreme adaptation strategy to excessive metal(loid) concentrations in the soil is rather rare, especially in comparison to Ni, which is hyperaccumulated by >70 % of all known trace element hyperaccumulator species (Reeves *et al.*, 2018). Among known Tl hyperaccumulator plant species, *Biscutella laevigata* and *Iberis linifolia* subsp. *intermedia* (synonym *I. intermedia*; POWO, 2024) have been studied the most intensively to date (LaCoste *et al.*, 1999; Fellet *et al.*, 2012; Reeves *et al.*, 2018; Corzo Remigio *et al.*, 2022*a*, *b* ). *Biscutella laevigata* growing in nature at a former Pb/Zn mine site in the Italian Alps can accumulate up to 32 700 mg kg^−1^ Tl in leaves (Fellet *et al.*, 2012; Pošćić *et al.*, 2015), whereas *I. linifolia* subsp. *intermedia* can accumulate up to 13 400 mg kg^−1^ Tl in its leaves when grown on Tl-spiked soil (Scheckel *et al.*, 2004). More recently, the highest ever concentration of Tl in a plant was recorded in *Silene latifolia* from the Allchar site with 79 200 mg kg^-1^ Tl in its leaves (Jakovljević *et al.*, 2025).

Hyperaccumulation of Tl has reported in three *Viola* taxa from the Allchar site with mean foliar Tl concentrations of 23 900, 7060 and 4650 mg kg^−1^ in *Viola arsenica*, *V. allchariensis* and *V. tricolor* subsp. *macedonica*, respectively (Jakovljević *et al.*, 2023). Arsenic hyperaccumulation is mainly associated with ferns of the genus *Pteris*, with *P. vittata* being the best studied among them (Ma *et al.*, 2001; Chen *et al.*, 2002; Wei *et al.*, 2020). Similar As concentrations have also been found in the fronds of several other *Pteris* taxa, such as *P. umbrosa*, *P. longifolia* and *P. cretica* (Zhao *et al.*, 2002), as well as in another fern, *Pityrogramma calomelanos* (Corzo-Remigio *et al.*, 2023). The *Viola* taxa from Allchar are not As hyperaccumulators, with *V. arsenica* having the highest concentrations with up to 381 mg kg^−1^ As (Jakovljević *et al.*, 2023).

Considering the co-occurrence in some deposits, co-accumulation of As and Tl has also been observed, such as in *P. vittata*, with an even higher bioaccumulation factor for Tl than for As (Wei *et al.*, 2020). Recently, this phenomenon was found in *Minuartia verna* and *Viola* taxa from the Allchar site (Bačeva Andonovska *et al.*, 2021; Jakovljević *et al.*, 2024). The leaves were the most important storage organ for Tl in *V. tricolor* subsp. *macedonica* and *V. allchariensis*, whereas for As the highest concentrations were found in the roots and in the seeds, respectively (Bačeva Andonovska *et al.*, 2021), suggesting different mechanisms of accumulation and tolerance between these two elements in the *Viola* species from the Allchar site. High accumulation of Tl in the leaves of *Viola* species from the Allchar area suggests potential for use in phytoremediation, which is of particular interest because there are few known hyperaccumulators of this element (Reeves *et al.*, 2018), but they have very low biomass production.

Given the co-accumulation of Tl and As and assuming different pathways for uptake and preferential sites of deposition, we aimed to determine the distribution of these elements in the two *Viola* species, *Viola tricolor* subsp. *macedonica* and *Viola arsenica*, which are among the strongest (hyper)accumulators. A previous study on the metallophyte *Viola* species from the Allchar site has shown that extremely high Tl in all three *Viola* taxa (*V. tricolor* subsp. *macedonica*, *V. allchariensis* and *V. arsenica*) and also As in *V. arsenica*, is endogenous and not the result of contamination, whereas As in *V. tricolor* subsp*. macedonica* and *V. allchariensis* was strongly affected by contamination (Jakovljević *et al.*, 2023). To exclude any possibility of contamination, we cultivated *V. tricolor* subsp. *macedonica* and *V. arsenica* in hydroponics and dosed the plants with As and Tl. This allowed us to perform synchrotron-based micro-X-ray fluorescence (µXRF) analysis on hydrated specimens to determine tissue- and cellular-level distribution of As and Tl.

## MATERIALS AND METHODS

### Plant culture conditions

**Fig. 1.**
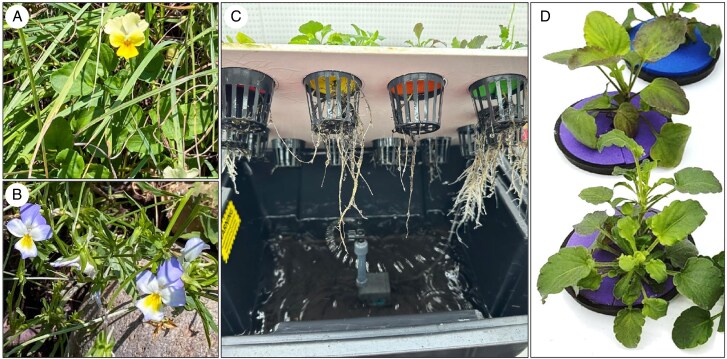
*Viola* species in their natural habitat and hydroponics. (A) *Viola arsenica* at the Allchar site. (B) *Viola tricolor* subsp. *macedonica* at the Allchar site. (C) Hydroponic set-up. (D) *Viola arsenica* (top) and *V. tricolor* subsp. *macedonica* (bottom) in hydroponics cultivation.

*Viola tricolor* subsp. *macedonica* and *V. arsenica* (Fig. 1) seeds were collected from natural populations growing at the Allchar site. The seeds were sown in trays on moist perlite, then placed in the fridge for 1 month to break dormancy. When the first seeds started to germinate, the trays were transferred to a growth chamber with a constant temperature of 23 °C and a photoperiod of 16 hr-8 hr light–dark. Two-week-old seedlings were transferred to Nutriculture20 aeroponic systems (Nutriculture Ltd, Skelmersdale, UK) filled with half-strength Hoagland solution. Plants were cultivated for 6 weeks prior to harvest: the first week with normal Hoagland solution, and from the second week with Tl and As treatment supplied in the form of Tl(I)NO_3_ and Na_2_HAsO_4_ at the following concentrations: 0 + 0 µM Tl + As (control), 1 + 0.5 µM Tl + As, 2 + 1 µM Tl + As and 4 + 2 µM Tl + As. The nutrient solution was maintained at a pH of 5.8 ± 0.1 and replaced fully weekly. Plants were grown for 6 weeks under the same temperature and light conditions as specified above prior to synchrotron and elemental analysis (Fig. 1C, D).

### Elemental analysis of plant and soil material samples

Prior to chemical analysis, the plant material was dried in a dehydrating oven at 60 °C for ≥48 hr. The plant material was then ground to a fine powder (<200 µm) in an impact mill (IKA TubeMill 100 Control). A subsample of 0.5 g was placed in custom XRF sample holders and covered with a 6.0 µm thin polypropylene film (Chemplex Industries Inc.) for XRF analysis. The XRF analysis of the plant powdered material was performed using a Z-Spec JP500 instrument (Z-Spec Inc.) (Kahlon *et al.*, 2024; Zhang *et al.*, 2025). This instrumentation uses monochromatic X-ray fluorescence excitation at 17.48 keV to analyse elements *Z* = 13 (Al) to *Z* = 39 (Y) on the K-lines and up to *Z* = 92 (U) on the L-lines with optimal sensitivity for the elements from Ca to Se and Hg–Tl–Pb. The limits of detection are reported by Z-Spec Inc as: Hg, 0.015 mg kg^−1^; Pb, 0.042 mg kg^−1^; As, 0.035 mg kg^−1^; and Se, 0.05 mg kg^−1^. Samples were analysed for 30 s in plant mode. Quality controls included NIST SRM 1570a (trace elements in spinach leaves) and NIST SRM 1573a (trace elements in tomato leaves).

### Synchrotron µXRF experiments

The experiments were conducted at PETRA III, a 6 GeV synchrotron radiation source that is part of DESY (Deutsches Elektronen-Synchrotron), specifically at the hard X-ray microprobe undulator beamline P06 (Boesenberg *et al.*, 2016). More information on instrumental parameters and set-up is given in van der Ent *et al.* (2023). The incident X-ray energy was 18 keV for the entire experiment. Depending on the elemental concentrations in the sample and the required resolution, Kirkpatrick-Baez (KB) mirrors and prefocusing compound refractive lenses (CRLs) were used to focus the beam down to either 830 nm × 430 nm (*horizontal* × *vertical*) with a flux of 3.6 × 10^10^ photons s^−1^ or 3.6 µm × 920 nm (*horizontal* × *vertical*) with a flux of 1.25 × 10^11^ photons s^−1^. For XRF detection, a Vortex ME4 in 45° geometry and a prototype 16-element Silicon Drift Ardesia Detector in 315° geometry (Utica *et al.*, 2021) with Xspress 3 pulse processors were used. The plant organs and tissues were held between two sheets of Ultralene thin film (4.0 μm thickness) stretched over a Perspex frame to prevent dehydration during the measurement. The tissue samples were sectioned using a ‘dry knife’ method to avoid elemental deportment and immediately mounted between the two layers of Ultralene thin film.

### Data processing and statistical analyses

The XRF data were processed with a non-linear least-squares fitting as implemented in PyMCA (Solé *et al.*, 2007). To conduct elemental calibration, thin film deposited foils containing elements with XRF emission lines close to the energy range of interest were measured (Micromatter Technologies Inc., Canada). The elemental calibrations for elements not covered by the foils were calculated using the results of the foil measurements in combination with elemental parameters from xraylib (Schoonjans *et al.*, 2011). This resulted in 32-bit .tiff files, with pixel values corresponding to the areal density of each element (in micrograms per centimetre squared). ImageJ (Schneider *et al.*, 2012) was used to create the figures, changing LUT to ‘Fire’, adjusting the maximum values, and using the ‘Calibration’ tool to add concentration bars and length scales. The Kruskal–Wallis test followed by Dunn’s test were performed on elemental data to determine significant differences using R software v.4.4.1.

## RESULTS

### Tolerance and accumulation of arsenic and thallium in *Viola* in hydroponics

Two *Viola* taxa, *V. tricolor* subsp. *macedonica* and *V. arsenica*, were dosed with a mixture of increasing concentrations of As and Tl, with different responses in the roots and shoots. Both species accumulate more As in the roots, with higher prevailing As concentrations in *V*. *arsenica*, except in the root from the control and in the shoot from the treatment with 0.5 µM As (Table 1). The highest As concentrations were found in the plants treated at 2 µM As, with a generally linear response to the applied As concentration in *V. arsenica*. The concentrations of Tl in the roots of *V*. *tricolor* subsp. *macedonica* increased with the dose level, reaching a mean value of 1110 mg kg^−1^ when treated with 4 µM Tl, with much lower Tl concentrations in *V. arsenica*. Much more uniform concentrations were observed in the shoots, with the concentrations in *V. arsenica* reaching higher values, except for the highest Tl dose applied. For As, no statistically significant differences were found between the two species at the lowest As dose rates, whereas the opposite was found for Tl, whereby lower dose rates led to significant differences between the species (Table 1). The dry weight of the plants varied within a narrow range, with no statistically significant differences between the samples and with the highest values observed in the control samples of both plant species. The concentrations of all analysed elements, other than As and Tl, are given in Supplementary Data Table S1.

**Table 1.** Plant dry weight (in grams per plant) and As and Tl concentrations (in milligrams per kilogram) in roots and shoots of *Viola arsenica* and *V. tricolor* subsp. *macedonica* grown under different treatments. Treatment concentrations are expressed in μM of Tl + As.

Treatment (Tl + As)	Taxon	Plant organ	DW	As	Tl
0 + 0	*V. arsenica*	Root	0.041 ± 0.005^a^	1.36 ± 0.08^a^	0.17 ± 0.09^a^
1 + 0.5	*V. arsenica*	Root	0.041 ± 0.004^a^	2.06 ± 0.2^a^	6.86 ± 0.2^b^
2 + 1	*V. arsenica*	Root	0.039 ± 0.001^a^	3.08 ± 0.2^ab^	10.2 ± 2^c^
4 + 2	*V. arsenica*	Root	0.038 ± 0.002^a^	4.9 ± 0.08^b^	35.6 ± 6^c^
0 + 0	*V. tricolor* subsp*. macedonica*	Root	0.083 ± 0.009^a^	1.87 ± 0.8^a^	0.03 ± 0.04^a^
1 + 0.5	*V. tricolor* subsp*. macedonica*	Root	0.071 ± 0.007^a^	1.62 ± 0.03^a^	146 ± 10^b^
2 + 1	*V. tricolor* subsp*. macedonica*	Root	0.075 ± 0.002^a^	< LOD^b^	1100 ± 50^c^
4 + 2	*V. tricolor* subsp*. macedonica*	Root	0.075 ± 0.008^a^	2.37 ± 1^b^	1110 ± 50^c^
0 + 0	*V. arsenica*	Shoot	0.98 ± 0.09^a^	1.90 ± 0.5^a^	6.24 ± 2^a^
1 +0.5	*V. arsenica*	Shoot	0.98 ± 0.09^a^	0.81 ± 0.02^a^	58.1 ± 6^b^
2 + 1	*V. arsenica*	Shoot	0.94 ± 0.007^a^	1.19 ± 0.2^a^	302 ± 60^c^
4 + 2	*V. arsenica*	Shoot	0.93 ± 0.05^a^	2.80 ± 0.08^b^	222 ± 70^c^
0 + 0	*V. tricolor* subsp*. macedonica*	Shoot	1.4 ± 0.2^a^	0.87 ± 0.1^a^	0.69 ± 0.1^a^
1 + 0.5	*V. tricolor* subsp*. macedonica*	Shoot	1.2 ± 0.1^a^	0.84 ± 0.3^a^	28.1 ± 1^b^
2 + 1	*V. tricolor* subsp*. macedonica*	Shoot	1.23 ± 0.03^a^	< LOD^b^	201 ± 10^c^
4 + 2	*V. tricolor* subsp*. macedonica*	Shoot	1.2 ± 0.1^a^	0.637 ± 0.5^a^	297 ± 20^c^

Values are expressed as mean ± s.d. of three replicates. LOD denotes below the limit of detection. Different letters indicate significant differences among treatments for a given species and plant part, after the Kruskal–Wallis test followed by Dunn’s test (*P* < 0.05, *n* = 3).

### Distribution of arsenic and thallium in *Viola* roots and stems

**Fig. 2.**
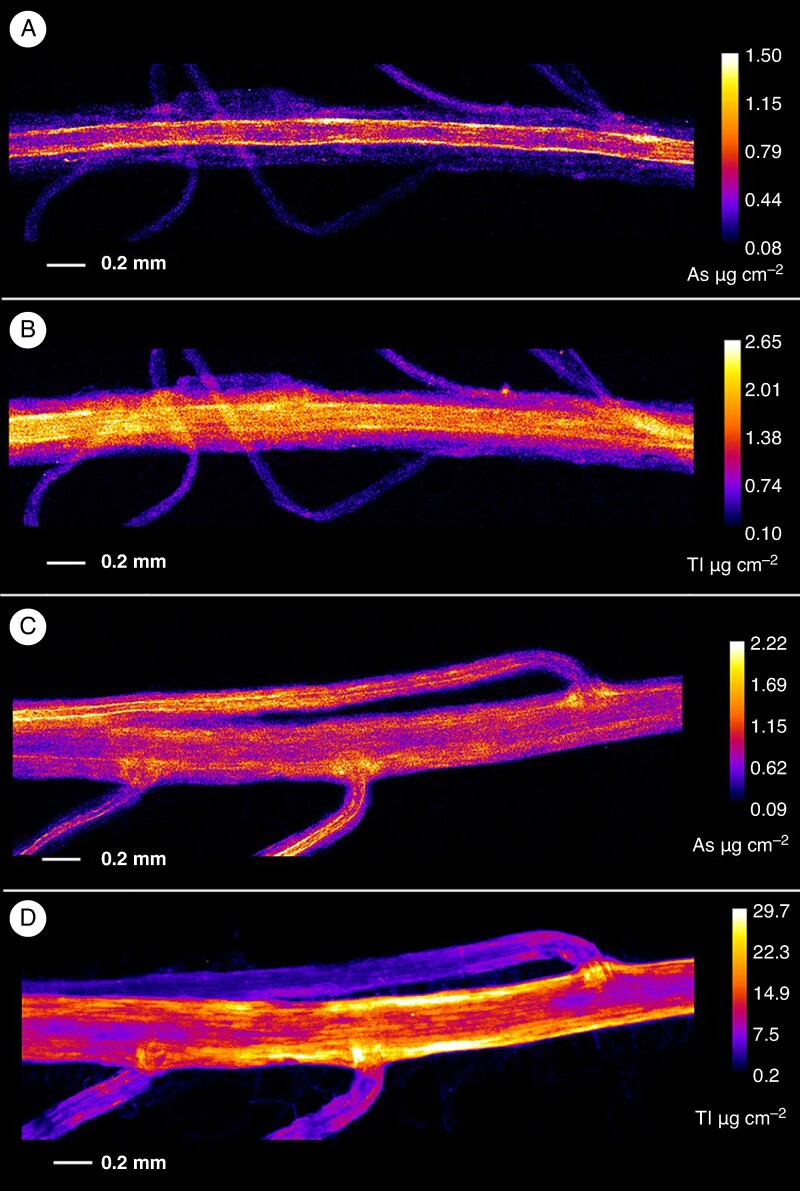
Root arsenic and thallium µXRF elemental maps. (A, B) Elemental distribution in *Viola tricolor* subsp. *macedonica*. (C, D) Elemental distribution in *Viola arsenica*. The elemental image was acquired with a 5 μm (for A and B) or 4 μm (for C and D) step size with 5 ms dwell per pixel. Length scales and concentrations ramp are included for each specimen.

**Fig. 3.**
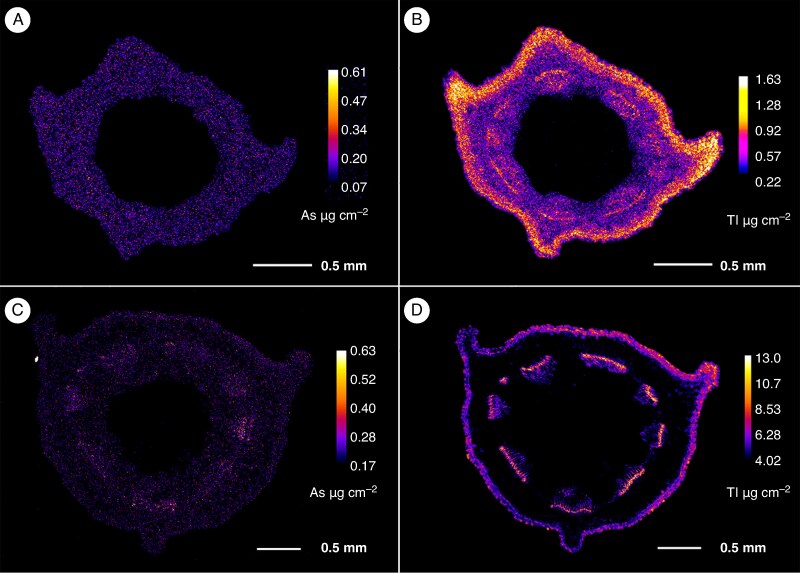
Stem (cross-sections) arsenic and thallium µXRF elemental maps. (A, B) Elemental distribution in *Viola tricolor* subsp. *macedonica*. (C, D) Elemental distribution in *Viola arsenica*. The elemental image was acquired at a 6 μm step size with 5 ms dwell per pixel. Length scales and concentrations ramp are included for each specimen.

**Fig. 4.**
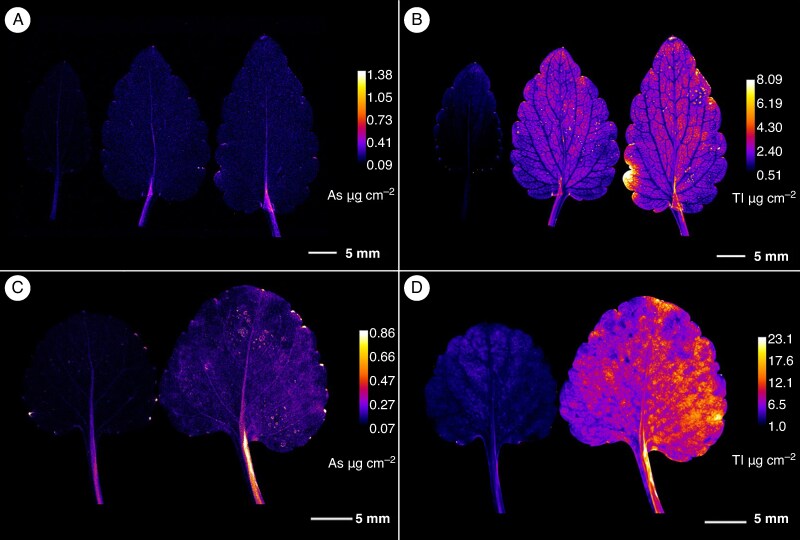
Arsenic and thallium µXRF elemental maps of leaves. (A, B) Elemental distribution in *Viola tricolor* subsp. *macedonica*. (C, D) Elemental distribution in *Viola arsenica*. The elemental images were acquired at a 25 μm (for A and B) or 20 μm (for C and D) step size with 5 ms dwell per pixel. Length scales and concentration ramps are included for each specimen.

**Fig. 5.**
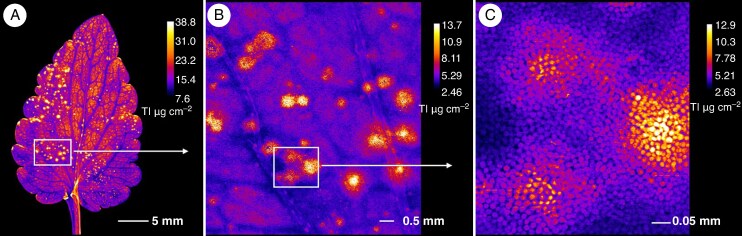
Details of thallium hotspots in *Viola tricolor* subsp. *macedonica* leaves. (A) Low-resolution overview scan. (B) Medium-resolution scan of central part of leaf. (C) High-resolution scan of area. The elemental images were acquired at a 20 μm (A), 5 μm (B) or 1.5 μm (C) step size with 5 ms dwell per pixel. Length scales and concentration ramps are included for each specimen.

**Fig. 6.**
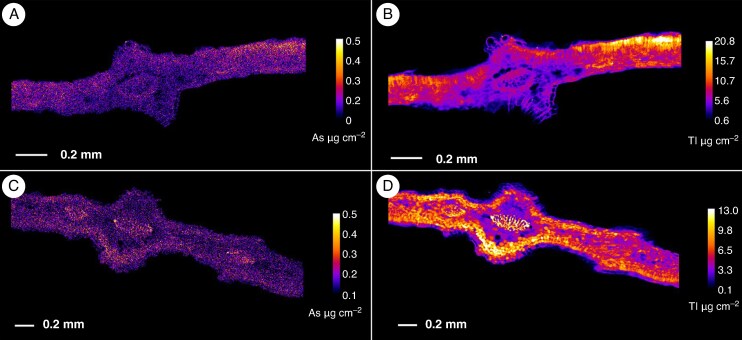
Arsenic and thallium µXRF elemental maps of leaves in cross-section. (A, B) Elemental distribution in *Viola tricolor* subsp. *macedonica*. (C, D) Elemental distribution in *Viola arsenica*. The elemental images were acquired at a 3 μm (for A and B) or 4 μm (for C and D) step size with 5 ms dwell per pixel. Lngth scales and concentration ramps are included for each specimen.

The µXRF elemental maps of *V. tricolor* subsp. *macedonica* and *V. arsenica* hydrated plant organs and tissues are shown in Figs 2–6. Different patterns of As and Tl accumulation were found in the root tissues of *V. tricolor* subsp. *macedonica*. The highest As concentrations were observed in the epidermis, whereas Tl appeared to be more evenly distributed, particularly in the cortex, with lower concentrations in the epidermis (Fig. 2A, B). The epidermis was also the main site for the localization of As and Tl in the roots of *V. arsenica*, for As in both the main and the lateral roots (Fig. 2C, D). The cross-sections of the stems of the two analysed *Viola* species showed a predominant Tl accumulation in the vascular cambium, with an enrichment also observed in the epidermal cells (Fig. 3). A similar pattern was observed for As, especially in *V*. *arsenica*. The enrichment of both As and Tl was shown to be higher in *V*. *arsenica*, with higher concentrations in the stem cortex.

### Distribution of arsenic and thallium in *Viola* leaves and petioles

Analysis of *V. tricolor* subsp. *macedonica* leaves revealed a low accumulation of As in the petiole and, to a lesser extent also in the midrib of the older leaves (Fig. 4). The enrichment of As in the leaf base is likely an artefact of over-laying (folded) plant tissues than true accumulation. Thallium accumulation in the leaves was pronounced with most of the Tl being localized in the central part of the lamina, especially in the mature leaves, but there was also strong accumulation of Tl in the petioles (Figs 4 and 5). The depletion of Tl in the vascular system in *V. tricolor* subsp. *macedonica*, as shown in Fig. 5, was also observed in leaf cross-sections, where accumulation was also found in the mesophyll, especially in the palisade (Fig. 6). The distribution of As and Tl in the leaves of *V. arsenica* is somewhat similar to that of *V. tricolor* subsp. *macedonica* (Fig. 4). Higher concentrations of both elements were found in the older leaves, with a more pronounced enrichment of Tl, which, unlike in the leaves of *V. tricolor* subsp. *macedonica*, was also found in the vessels, as indicated by some enrichment of the veins and veinlets. This accumulation pattern was also observed in the leaf cross-section, but with enrichment in the epidermis and in the cells of the palisade (Fig. 6).

## DISCUSSION

Two metallophyte *Viola* taxa from the Allchar site, which is naturally extremely enriched in Tl and As, were studied experimentally for the first time to assess the distribution of As and Tl and to infer tolerance mechanisms responsible for (hyper)accumulation. The enrichment patterns of these two elements differed to some extent, with both taxa accumulating high concentrations of Tl, albeit with opposite trends. In contrast to the strong exclusion in *V. tricolor* subsp. *macedonica* and the predominant accumulation in the roots, in the shoot of *V. arsenica*, Tl concentrations were higher in all treatments. Both root and shoot accumulation of Tl proved to be dose dependent, as was also observed in *S. latifolia*, another hyperaccumulator of this element (Regini *et al.*, 2024). In addition to the preferential accumulation of Tl in the shoots, similarly to *S. latifolia*, *V. arsenica* had a high Tl tolerance that did not reduce K uptake, as can be seen in the Tl concentrations at the highest dose rate (Supplementary Data Table S1). Remarkably, these Tl hyperaccumulator species are able to maintain normal growth when growing on what are clearly extremely toxic soils (Regini *et al.*, 2024). In contrast, *V. tricolor* subsp. *macedonica* had a significant decrease in K concentrations at the highest Tl dose rate, indicating a possible antagonism between these two elements and a lower level of Tl tolerance. For As, both of the *Viola* species are evidently excluders of this element. The retention of As in the roots is characteristic for excluder species and prevents translocation to the shoot to avoid toxicity (de Freitas-Silva *et al.*, 2016). In the roots of both species, the concentrations of P, S and K were significantly higher under the highest As and Tl treatments compared with control plants. This might be because the increase in P concentrations could reflect competition between arsenate and phosphate for uptake *via* phosphate transporters and upregulation of the latter. In addition, higher S concentrations might indicate activation of detoxification mechanisms, such as synthesis of phytochelatins, glutathione or metallothioneins (Seregin and Kozhevnikova, 2023).

Mature leaves were shown to be one of the main deposition sites for Tl, especially in *V. arsenica*, which is in line with the (hyper) accumulator strategy of the species. A similar pattern was found in green cabbage, where 80% of the concentrations of this element (Jia *et al.*, 2013) and ≤95% in *Iberis linifolia* subsp. *intermedia* (Scheckel *et al.*, 2004) are accumulated in this plant organ. Thallium localization in the leaves was also found in the hyperaccumulators *B. laevigata* and *S. latifolia* (Corzo Remigio *et al.*, 2022*a*, *b*).

The accumulation of Tl in the leaves of *V. tricolor* subsp. *macedonica* was mainly associated with the leaf blade, especially in the central parts, extending towards the leaf margins, whereas the conspicuous depletion in the veins and veinlets suggests that the vessels are not the main deposition sites for Tl in the leaves. In fact, the high-resolution scans suggest that Tl excretion after it reaches (and concentrates around) the stomata *via* the transpiration stream is a possible detoxification mechanism. Likewise, in *B. laevigata*, another Tl hyperaccumulator, the distribution of Tl in leaves follows the transpiration stream (Corzo Remigio *et al.*, 2022*a*), but also of Ni in lemongrass (Pandey *et al.*, 2020) and in *Noccaea japonica* (synonym *Thlaspi japonicum*), an endemic Ni hyperaccumulator from Japan (Mizuno *et al.*, 2003) and in the non-accumulator *Thlaspi arvense* (Nascimento *et al.*, 2021), whereas in *Brassica* cultivars abundant Tl-enriched crystals were also found in guard cells of the foliar stomata (Corzo-Remigio *et al.*, 2024). It might be that Tl translocates *via* the apoplast towards the stomatal region, where it accumulates owing to the absence of plasmodesmata in guard cells, as found for As in mung bean leaves (Gupta and Bhatnagar, 2015).

In contrast to *V. tricolor* subsp. *macedonica*, accumulation of Tl and As was found in the vascular system in the leaves of *V. arsenica*, especially in the petiole and in the proximal parts of the leaf. The vascular system also seems to be the main site for the localization of Tl in *I. linifolia* subsp. *intermedia* (Scheckel *et al.*, 2004) and in *S. latifolia*, one of the strongest hyperaccumulators of Tl, where high concentrations of this element were found mainly in the leaf veins and midrib (Corzo Remigio *et al.*, 2022*b*). Localization of As within the vascular bundles was also found in *Pteris vittata* (Kitajima *et al.*, 2008) and *Lonicera japonica* (Qiao *et al.*, 2023). Considering that the role of vascular bundles is primarily for transport, but also as a mechanical support, and not directly for the process of photosynthesis, the accumulation of As in this area could represent an adaptation strategy of the plant.

### Conclusion

The spatial enrichment patterns of As and Tl in *V. arsenica* and *V. tricolor* subsp. *macedonica* differed to some extent, with root accumulation of As in both taxa and opposing trends in the accumulation of Tl. Synchrotron-based μXRF analysis revealed a predominant deposition of Tl in mature leaves of *V. arsenica* with enrichment in the vascular system, especially in proximal leaf parts. In *V*. *tricolor* subsp. *macedonica*, besides retention in the roots, the exudation of excess Tl through the stomata could be a potential mechanism for survival under Tl toxic conditions.

## Supplementary Material

mcaf166_Supplementary_Data

## Data Availability

The data that support this study will be shared upon reasonable request to the corresponding author.

## SUPPLEMENTARY DATA

Supplementary data are available at *Annals of Botany*online and consist of the following. Table S1: plant elemental concentrations (in milligrams per kilogram) in roots and shoots of *Viola arsenica* and *V. tricolor* subsp. *macedonica* grown under different treatments. Values are expressed as the mean ± s.d. of three replicates.
