# Supplementary material for: Surviving Allchar: arsenic and thallium tolerance and distribution in Viola metallophytes
Source: Ann Bot. 2025 Jul 19;136(7):1515–24. doi: 10.1093/aob/mcaf166 (PMC12718056; doi:10.1093/aob/mcaf166)
Supplement: mcaf166_Supplementary_Data [file mcaf166_supplementary_data.docx]

**Suppl Table S1.** Plant elemental concentrations (mg kg^-1^) in roots and shoots of *Viola* *arsenica* and *V*. *tricolor* subsp. *macedonica* grown under different treatments. Values are expressed as mean ± standard deviation of three replicates.

| **treatment** | **organ** | **species** | **P** | **S** | **Cl** | **K** | **Ca** | **Cr** | **Mn** | **Fe** | **Cu** | **Zn** | **Br** |
| --- | --- | --- | --- | --- | --- | --- | --- | --- | --- | --- | --- | --- | --- |
| 0+0 | root | *V. arsenica* | 3370 ± 827 | 4100 ± 369 | 405 ± 49.3 | 2350 ± 129 | 6180 ± 996 | 3.12 ± 0.728 | 133 ± 49.9 | 383 ± 11 | 122 ± 17.6 | 16.9 ± 1.57 | 10.2 ± 0.940 |
| 0+0 | root | *V. tricolor subsp. macedonica* | 5120 ± 97.5 | 7320 ± 449 | 2320 ± 170 | 16900 ± 200 | 8610 ± 873 | 1.82 ± 0.693 | 326 ± 57.4 | 404 ± 65.5 | 141 ± 35.6 | 56.9 ± 4.19 | 16.3 ± 2.45 |
| 1+0.5 | root | *V. arsenica* | 5250 ± 722 | 7570 ± 394 | 763 ± 95.8 | 46400 ± 475 | 7430 ± 856 | 3.31 ± 1.53 | 203 ± 7.25 | 919 ± 83.8 | 466 ± 60.3 | 32.4 ± 0.622 | 23.5 ± 2.14 |
| 1+0.5 | root | *V. tricolor subsp. macedonica* | 6600 ± 391 | 6190 ± 343 | 8960 ± 628 | 62300 ± 1570 | 4750 ± 207 | 0.077 ± 0.045 | 1680 ± 123 | 256 ± 4.13 | 53.6 ± 2.33 | 51.9 ± 5.64 | 34.8 ± 2.04 |
| 2+1 | root | *V. arsenica* | 6890 ± 720 | 7180 ± 161 | 1290 ± 43.2 | 8410 ± 674 | 7020 ± 830 | 1.22 ± 0.184 | 289 ± 31.6 | 683 ± 67.1 | 388 ± 22.0 | 50.3 ± 1.24 | 21.5 ± 0.523 |
| 2+1 | root | *V. tricolor subsp. macedonica* | 9360 ± 532 | 12300 ± 677 | 3820 ± 80 | 60200 ± 1170 | 6410 ± 220 | 0.107 ± 0.105 | 2830 ± 32 | 365 ± 13.1 | 98 ± 5.71 | 84.3 ± 2.53 | 23.3 ± 0.665 |
| 4+2 | root | *V. arsenica* | 8060 ± 509 | 8340 ± 98.5 | 1540 ± 615 | 11200 ± 3700 | 9000 ± 301 | 2.36 ± 0.240 | 473 ± 189 | 776 ± 13.9 | 310 ± 2.2 | 49.4± 5.05 | 23.6 ± 0.028 |
| 4+2 | root | *V. tricolor subsp. macedonica* | 8130 ± 867 | 12400 ± 377 | 2010 ± 126 | 29800 ± 2430 | 7390 ± 357 | 1.32 ± 0.071 | 1360 ± 49 | 657 ± 24.5 | 248 ± 8.55 | 60.7 ± 1.63 | 24.0 ± 2.45 |
| 0+0 | shoot | *V. arsenica* | 2880 ± 405 | 3290 ± 77 | 4880 ± 252 | 30100 ± 525 | 7410 ± 748 | 0.025 ± 0.035 | 90.5 ± 3.42 | 73.7 ± 3.79 | 11.2 ± 0.127 | 28.7 ± 1.23 | 35.4 ± 0.594 |
| 0+0 | shoot | *V. tricolor subsp. macedonica* | 5200 ± 819 | 4750 ± 45.6 | 4390 ± 100 | 32100 ± 656 | 8920 ± 60.9 | 0.07 ± 0.099 | 138 ± 11.2 | 162 ± 21.4 | 15.5 ± 0.742 | 61 ± 3.01 | 41.1 ± 0.58 |
| 1+0.5 | shoot | *V. arsenica* | 2350 ± 436 | 2830 ± 158 | 464 ± 911 | 23000 ± 1350 | 7290 ± 1390 | 0.58 ± 0.042 | 142 ± 11.4 | 65.0 ± 4.04 | 10.5 ± 0.148 | 28.3 ± 0.064 | 39.0 ± 7.35 |
| 1+0.5 | shoot | *V. tricolor subsp. macedonica* | 5720 ± 439 | 420 ± 178 | 4460 ± 93.1 | 49500 ± 560 | 9910 ± 230 | 0.29 ± 0.017 | 254 ± 19.1 | 95.8 ± 6.96 | 8.95 ± 0.080 | 38.3 ± 2.48 | 41.1 ± 0.741 |
| 2+1 | shoot | *V. arsenica* | 1930 ± 44.1 | 3100 ± 138 | 4070 ± 130 | 22700 ± 1250 | 6320 ± 161 | 0.27 ± 0.382 | 104 ± 6 | 64.9 ± 3.83 | 8.57 ± 0.120 | 21.2 ± 0.636 | 30.0 ± 1.15 |
| 2+1 | shoot | *V. tricolor subsp. macedonica* | 4250 ± 409 | 5070 ± 268 | 3200 ± 189 | 53000 ± 1980 | 847 ± 400 | 0.003 ± 0.006 | 296 ± 10.9 | 71.8 ± 0.990 | 6.15 ± 0.296 | 48.9 ± 1.40 | 38.39 ± 0.890 |
| 4+2 | shoot | *V. arsenica* | 2490 ± 863 | 3390 ± 232 | 4350 ± 282 | 26100 ± 355 | 8920 ± 732 | 0.13 ± 0.184 | 154 ± 0.721 | 86.7 ± 2.15 | 10.6 ± 1.22 | 25.2 ± 2.07 | 33.7 ± 2.46 |
| 4+2 | shoot | *V. tricolor subsp. macedonica* | 3340 ± 74.5 | 46407 ± 173 | 27407 ± 212 | 49 ± 269 | 720± 428 | 0.2 ± 0.2 | 224 ± 7.84 | 68.8 ± 1.51 | 6.23 ± 0.276 | 33.0 ± 0.59 | 35.2 ± 1.376 |
